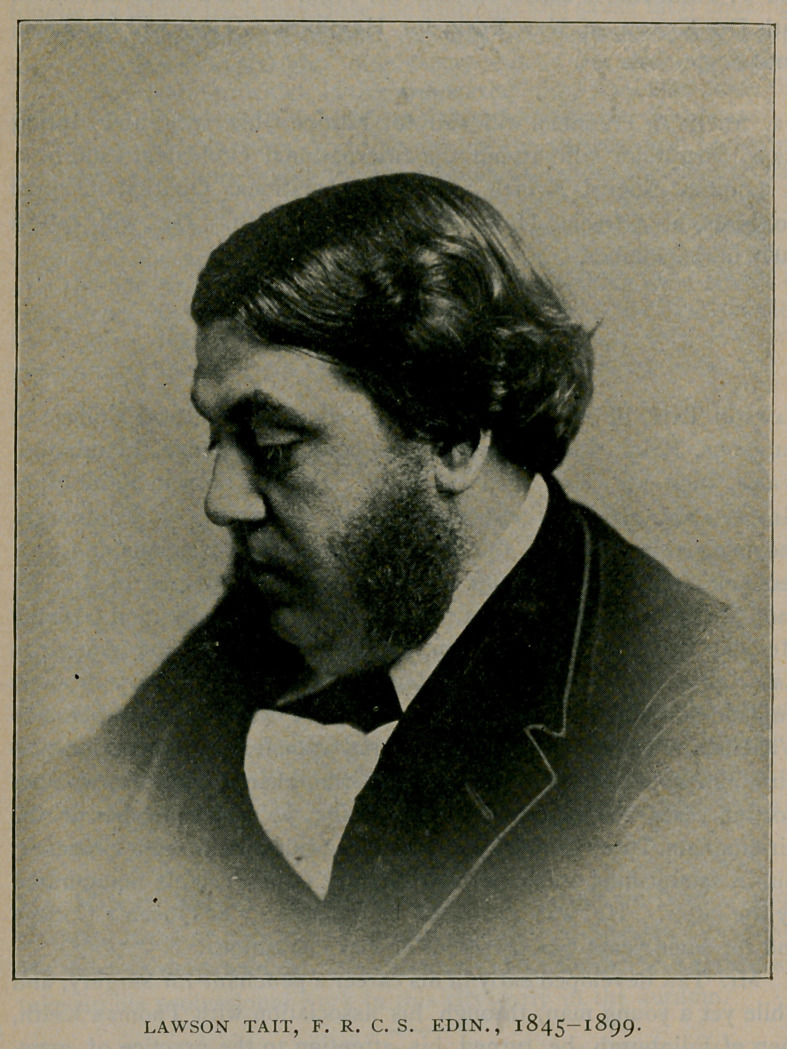# Lawson Tait

**Published:** 1899-07

**Authors:** 


### OBITUARY.

LAWSON TAIT, of Birmingham, Eng., Fellow of the Royal College of Surgeons, Edin., died suddenly at his country residence, Llandudno, Wales, June 13, 1899, aged 54 years. He was born in Edinburgh, May 1, 1845, and was educated at the University of Edinburgh, becoming a licentiate of the Royal Colleges of Physicians and Surgeons of Edinburgh in 1866. He received the degree of LL. D. and M. D. (honoris causa) from Union University and Albany Medical College. He was an honorary Fellow of the American Association of Obstetricians and Gynecologists and a Fellow of many English societies. From 1867 to 1870 he was house surgeon to Wakefield hospital and in 1871, having transferred his residence to Birmingham he became surgeon to the Birmingham hospital for women. For ten years—namely, from 1875 to 1885, he was a member of the Birmingham Town Council and was a coadjutor of Joseph Chamberlain in establishing municipal and sanitary improvements inaugurated by the latter. He was professor of gynecology at Queen's College and for some years was president of that institution.

Mr. Tait developed early in his career a penchant for surgery, and while yet a young man, through his association with Thomas Keith, then of Edinburgh, he turned his attention to the practice of gynecology. Very soon his surgical dexterity was recognised and his marvelous success in abdominal surgery made him famous throughout the world. His published results attracted great attention, but the record of success was so phenomenal that their truthfulness was challenged. Mr Tait, however, was able in every instance to meet his critics with a carefully preserved record of the name, residence of the patient, date and place of operation and other data so minutely described as to overwhelm his opponents, who were often prompted to adverse criticism by mere jealousy.

LAWSON TAIT, F. R. C. S. EDIN., 1845–1899.

Mr. Tait's technic was so simple, his work so rapid and his recoveries so prompt that his clientèle soon became very large. He early discarded clamps and other instrumental methods in the management of the ovarian pedicle, substituting therefor a small ligature neatly tied and dropped into the abdomen. This method was criticised most severely, but it proved so successful that other operators soon began to adopt it. We merely mention this as one of the novel methods introduced by Mr. Tait—a number of which were original with him—all tending to simplicity and most of which have been adopted by other operators.

His clinics at Birmingham, both public and private, soon became thronged with medical visitors from all parts of the world, but especially from the United States, who were anxious to see the man and learn the secret of his success; and "7, The Crescent," became the Mecca of the pilgrims who would learn the methods of the new school of abdominal surgery. They fain would sit at the feet of the master and, we are sorry to add, that we fear in some instances they did him sad justice on their return to their native country.

Mr. Tait's career, although comparatively a short one, was full of absorbing interest and remarkable achievement, not the least of which was to attain a world-wide reputation in a provincial city, for he commanded a clientèle at one time beyond that of any metropolitan surgeon. He was a voluminous writer, a strong debater, and was able to maintain his own position with tongue or pen, even at the risk sometimes of using caustic, if not questionable, rhetoric. But for this he can be forgiven as the provocation was many times extreme.

Mr. Tait had many peculiarities, but the only serious blot upon his memory is that he should have become, in a certain sense, the medical champion of the antivivisectionists. All through his stormy career he was sustained by an accomplished and devoted wife, who, in her early widowhood, will receive the sympathy of a magnanimous profession.

Mr. Tait's loss to the surgical world is distinctively a great one and a thousand years hence his name will be quoted as among those, who, during the nineteenth century contributed something to the advancement of medical science.

The portrait we publish is from a photograph taken in 1887, which was an excellent likeness of him at the time.

## Figures and Tables

**Figure f1:**